# Increased expression of miR-224-5p in circulating extracellular vesicles of patients with reduced coronary flow reserve

**DOI:** 10.1186/s12872-022-02756-w

**Published:** 2022-07-18

**Authors:** Kreema James, Paulina Bryl-Gorecka, Björn Olde, Olof Gidlof, Kristina Torngren, David Erlinge

**Affiliations:** Department of Cardiology, Clinical Sciences, Biomedical Centre, Faculty of Medicine, Lund University, D12, Sölvegatan 17, 22362 Lund, Sweden

**Keywords:** Major adverse cardiac events, Coronary flow reserve, Micro-RNA, Extracellular vesicles, Hepatic, Cardiovascular disease

## Abstract

**Background:**

Endothelial and microvascular dysfunction are pivotal causes of major adverse cardiac events predicted by coronary flow reserve (CFR). Extracellular Vesicles (EVs) have been studied extensively in the pathophysiology of coronary artery disease. However, little is known on the impact of the non-coding RNA content of EVs with respect to CFR.

**Methods:**

We carried out a study among 120 patients divided by high-CFR and low-CFR to profile the miRNA content of circulating EVs.

**Results:**

A multiplex array profiling on circulating EVs revealed mir-224-5p (*p*-value ≤ 0.000001) as the most differentially expressed miRNA in the Low-CFR group and showed a significantly independent relationship to CFR. Literature survey indicated the origin of the miR from liver cells and not of platelet, leukocyte, smooth muscle or endothelial (EC) origin. A q-PCR panel of the conventional cell type-EVs along with hepatic EVs showed that EVs from liver cells showed higher expression of the miR-224-5p. FACS analysis demonstrated the presence of liver-specific (ASGPR-1+/CD14−) EVs in the plasma of our cohort with the presence of Vanin-1 required to enter the EC barrier. Hepatic EVs with and without the miR-224-5p were introduced to ECs in-vitro, but with no difference in effect on ICAM-1 or eNOS expression. However, hepatic EVs elevated endothelial ICAM-1 levels per se independent of the miR-224-5p.

**Conclusion:**

This indicated a role of hepatic EVs identified by the miR-224-5p in endothelial dysfunction in patients with Low CFR.

**Supplementary Information:**

The online version contains supplementary material available at 10.1186/s12872-022-02756-w.

## Background

CAD is often characterized by a multitude of mechanisms including low nitric oxide (NO) bioavailability as well as oxidative stress, collectively leading to endothelial as well as microvascular dysfunction [1–3]. Various factors contribute to the development of CAD including lifestyle, hypertension, diabetes as well as genomic and molecular causes.

Myocardial Infarction (MI) is the eventual result of acute CAD, affecting nearly 7 million people every year in developed countries and increasingly in developing countries [4, 5]. Survivors of MI are at high risk of developing further cardiac events within a year of the first event [6]. Major Adverse Cardiac Events (MACE) is used as a composite endpoint to assess the morbidity as well as mortality of survivors [7]. Coronary Flow Reserve (CFR) is an important prognostic indicator of MACE [8]. It is measured as the ratio of coronary blood flow at its maximum during adenosine infusion to when at rest. The higher the CFR ratio the lower the risk of MACE.

Extracellular vesicles (EVs) are 10–500 nm membrane bound structures that play a crucial role in cellular communication especially in association to various diseases. These particles carry a content of active molecules that may reflect the cardiovascular condition [9]. EVs of endothelial origin have been studied to predict future cardiac events [10]. Similarly, EVs of various origins have been found to play a crucial role in cardiovascular diseases [11]. Exosomal EVs have also been used as biomarkers for ischemic heart disease and studied in the context of cardio-repair [12]. Emerging research has shown that EVs transport micro-RNAs depending on the pathophysiology of the cells affecting multitude of functions in their target cells [13, 14].

Micro-RNAs are short RNA transcripts that demonstrate specific regulatory capabilities depending on the cell’s physiology as well as micro-environment. Research have brought to light the role of micro-RNAs in regulating various molecular pathways including those involving inflammation, apoptosis, proliferation among others by repressing translation mechanisms [15]. Micro-RNAs are 18–22 nucleotide non-coding RNAs that can bind to the mRNA, mediating translational repression either by inhibiting the binding to either 60 s or 40 s ribosomal subunits or by activation of the RNA-induced silencing complex (RISC complex). Several micro-RNA families have been shown to regulate pathogenesis of CAD and MI [16, 17].

It has been observed that cardio-specific miRs increase in circulation after MI [18]. Decrease in miR-24 has been observed in heart tissue after MI while miR-325, miR-21, miR-16, miR-375 have shown to increase [19]. Circulating miR-26a-1, miR-146a and miR-199a-1 have also been demonstrated as biomarkers for acute myocardial infarction [20]. miR-93 and miR-153 have shown to regulate homeostasis and an anti-inflammatory role by inhibiting cardiomyocyte apoptosis [21, 22]. EV bound miR-222 has been studied for its anti-inflammatory effects in target cells by reducing endothelial ICAM-1 an effect that is reduced under hyperglycemic conditions [23]. EV-derived miR-146a accelerates the development of atherosclerosis [24].

Although MiRs have been investigated in cardiovascular disease, there has not yet been a study involving the relationship of micro-RNA transported in circulating EVs in relation to CFR. Therefore, to understand the role of EV bound micro-RNAs in our cohort we have prepared micro-RNAs from circulating EVs isolated using a novel acoustic trapping technique [25] and performed a q-PCR based array profile. The study concerns understanding the origin of the EVs in the circulating plasma and the effect of its cargo specifically micro-RNA, in the pathophysiology of patients with low CFR.

## Methods

### Cohort design and sample collection

The PROFLOW cohort comprises of 619 patients with prior type-1 MI (> 3 months and < 5 years), all were identified in the Swedish Coronary Angiography and Angioplasty Registry (SCAAR) by the Department of Cardiology at Skåne University Hospital in Lund and Sahlgrenska University Hospital in Göteborg, Sweden. Coronary Flow Reserve (CFR) was measured as the ratio between the hyperemic and baseline flow velocity values at rest and after induction with adenosine (140ug/kg/min) in the identified and defined Left Anterior Descending artery (LAD) with 3.5‐MHz color Doppler in the interventricular sulcus in a modified 2‐chamber view [26]. The blood samples were collected from fasting patients in EDTA tubes. Plasma was obtained from the drawn blood samples by centrifugation within 30 min of drawing and stored at −80 °C. Informed written consent was undertaken in agreement with the the International Conference on Harmonization Good Clinical Practice guidelines. The study was reviewed and accepted by the institutional ethical committee at Gothenburg University.

For the study of circulating EVs we chose 120 patients from Lund, based on CFR; segregated into two groups based on a median CFR threshold of 2.9. The patients with CFR ≥ 2.9 were assigned to the High CFR group, whereas the patients with CFR ≤ 2.9 were assigned to the Low CFR group. The High CFR group was considered as the control. The handling of patient data and samples was conducted in accordance with the Declaration of Helsinki.

### EV isolation and enrichment

The bio-banked plasma samples (75 μl) were thawed at room temperature and diluted 1:5 in DPBS. Acoutrap (AcouSort AB, Lund, Sweden) an automated platform was used to isolate EVs with standing ultrasound waves; a novel method that has been previously described in detail and validated by TEM as well as NTA by us [27–29]. The diluted plasma was aspirated 25 μl/min into a capillary with pre-suspended seed particles (12 μm polystyrene beads, Sigma-Aldrich). The cloud of EVs is then washed and released with 50 μl of DPBS. The process was repeated thrice per sample for maximum enrichment of EVs making the final volume of collected EVs to be 150 μl. They were immediately lysed using 600 μl of Lysis Buffer (Lysis Buffer A, Human Plasma/Serum RNA purification kit, Norgen Biotek Corp.) for further processing.

### RNA extraction and assessment

Total RNA was extracted according to manufacturer’s instruction (Human Plasma/Serum RNA purification kit, Norgen Biotek Corp.) from the trapped samples and was stored at -20 °C until required. The quality and quantity of RNA was assessed by Eukaryote Total RNA Pico Assay on a Aligent 2100 Bioanalyzer system (SCIBLU, Lund, Sweden) using 5 μl of the total RNA.

### Real-time PCR based array

The micro-RNA content in the samples was explored with a Real-time PCR based array (TaqMan Advanced miRNA Human Serum/Plasma Card) in a 384-well microfluidic card with 190 unique miRNAs in duplicates. The RNA was amplified by uniform universal amplification to make cDNA prior to the qPCR as per the manufacturer’s instruction. The card also contained an endogenous control (miR-16-5p) as well as two exogenous controls (ath-miR-159a & cel-miR-39-3p). The assay was performed on the QuantStudio 12 k flex system. The expression of the miRNAs was determined by calculating the Relative fold change (2^-ddCT). The data was normalized using global mean in each sample.

### Statistical analysis

The relative fold change data of only those samples were considered where endogenous control had amplified while exogenous controls showed no amplification. The data was subjected to ROUT outliers test (Q = 1%), followed by Multiple t-test (one per row) for unpaired samples on the cleaned data, with False Discovery Rate approach (FDR, Benjamini, Krieger and Yekutieli). The discoveries were also analyzed for correlation with clinical data. We used simple logistic regression for clinical data in binary format. The statistical analysis was performed on GraphPad Prism (Version 8.2).

### Isolation of EVs from cell culture

Human Coronary Artery Smooth Muscle (Gibco), Human Coronary Artery Endothelial (Cell Applications Inc.) and HepG2 cells were cultured in M231-SMGS (Gibco), MesoEndo Cell Growth Medium with Growth factors (Cell Applications Inc.) and RPMI Medium 1640, respectively. Leukocytes were isolated according to the manufacturer’s instruction with Ficoll-Paque Plus (GE Healthcare) whereas platelets were isolated and activated according to the protocol by Landsberg et.al and optimized in-house for our experiments [30].

The cells were seeded with the required medium and incubated overnight. At 60–80% confluency, the cells were stimulated with TNF-alpha (10 ng/μl) to generate EVs whereas thrombin receptor agonist peptide-6 (TRAP) was used to generate EVs from platelet rich plasma (PRP). The supernatant was collected after 16–20 h post-stimuli and then centrifuged at 500 G, followed by 2500G for 10 min each and ultimately at 10,000G for 1 h 30 min at 4 °C. The vesicle pellet was resuspended in 500ul DPBS, followed by RNA extraction (Human Plasma/Serum RNA purification kit, Norgen Biotek Corp.) according to manufacturer’s protocol. Presence of miRNAs in the EVs was analyzed with real-time PCR (miRCURY LNA miRNA PCR Assay) and determined by relative fold change with U6 snRNA (miRCURY LNA miRNA PCR Assay) as the reference gene.

### Analysis of vesicle origin in plasma

To investigate if liver EVs are present in the circulation, selected samples from the ProFlow cohort were analyzed with flow cytometry (FCM) with an ApoGee A60 cytometer (Apogee Flow Systems, Hemel Hempstead, UK). The calibration of the instrument was performed on the basis of the protocol established previously for the characterization of acoustically trapped EVs for the same cohort [31] with size-calibrated silica beads (Apogee Mix, Apogee Flow Systems) and antibody (Ab) isotype controls: Alexa Fluor 488 mouse IgG1κ, PE mouse IgG1κ, FITC mouse IgG1κ, as well as Abs in DBPS. To remove Ab aggregates, the Abs were high-speed centrifuged at 20,000×*g* for 30 min at 4 °C. Patient plasma samples were diluted 1:200 with DPBS. 200 μl was further stained with 2 μl of asialoglycoprotein receptor 1 (ASGPR1) PE (liver derived EVs), for 30 min in RT and directly analyzed. To exclude monocyte derived EVs from the ASGPR1 + EV pool, a co-staining with CD14 FITC Ab was performed. To determine if liver EVs co-express vanin-1 (VNN1) on the surface, Vanin-1 FITC Ab was used for co-staining. Since CD14 was also FITC labelled, we chose to co-stain for ASGPR1 and Vanin-1 for the determination of vanin-1 containing hepatic EVs. Mouse isotype controls, mouse Abs: CD14 clone were purchased from BD Biosciences. Mouse Abs: ASGPR1 clone, rabbit isotype control FITC and rabbit Ab Vanin-1 clone Abs were purchased from manufacturer. The experiment was repeated in order to determine successful reproducibility.

### Analysis of ASGPR1 presence on liver cancer cell line vesicles

The vesicle pellet was resuspended in 500 μl DPBS. 200 μl of the suspension was diluted and stained with 2 μl ASGPR1 PE Ab for 30 min in RT and analyzed with an ApoGee cytometer using previously established EV gate.

### In-vitro transfer of EVs

HepG2 cells were transfected with mimic (100 nM, HMI0403) and inhibitor (50 nM, HSTUD0403) for miR-224-5p (MISSION® Sigma Aldrich) by nucleofection (Amaxa Nucleofector Kit, Lonza Biosciences) according to manufacturer’s recommendations. HepG2 cells were used as control. Simultaneously endothelial cells (approximately 5000 cells per well) were seeded in a 24-well plate and incubated overnight. EVs were generated from the HepG2 cells as stated before and added 100 μl per well to endothelial cells and incubated overnight at 37 °C in the required medium. The cells were harvested the following day for real-time validation. The EVs generated were also validated for surface markers ASGPR-1 and Vannin-1. Endothelial cells with EVs from HepG2 cells were used as positive control whereas cells without addition of EVs were kept as experimental control.

#### Analysing the role of hepatic EVs on endothelial cells

HCMVEC cells were subjected to EVs as described above and then assayed for proliferation using CCK-8 (Dojindo Laboratories) according to the manufacturer’s instructions. The cell density was determined using crystal-violet in reference to the assay by Grossi et al. [32] and optimized according to our experimental setup. HCMVEC cells were also analyzed for the expression levels of surface ICAM-1 after incubation with EVs for 24 h as described previous by Bryl-Gorecka et al. [31].

## Results

### EV micro-RNA profiling based on CFR

**Fig. 1:**
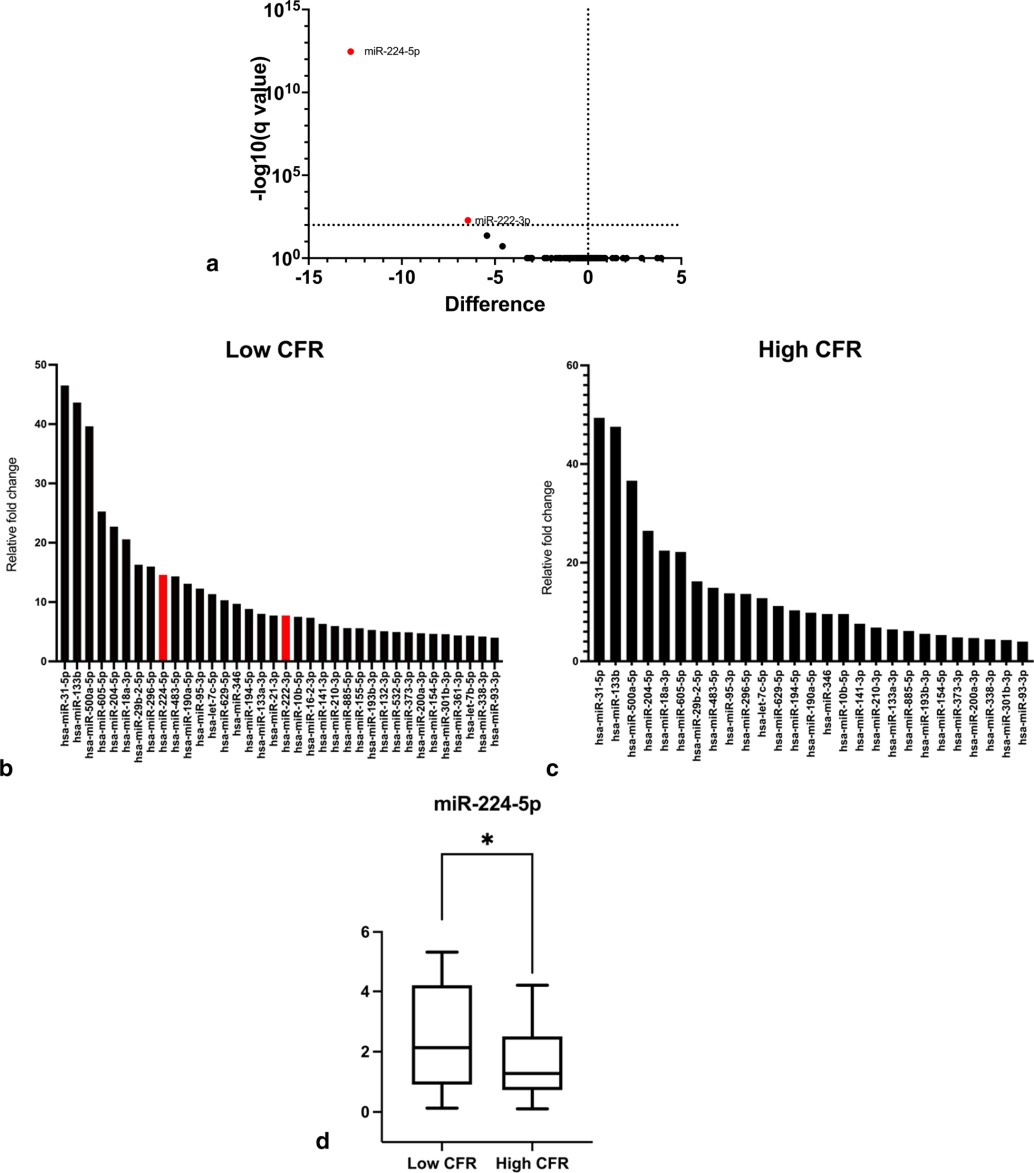
The EV-profiling led to the discovery of miR-224-5p and 222-3p in the low CFR group. **a** demonstrates the volcano plot describing the relevance of the miRs with respect to *p*-value and relative fold change. **b**, **c** are representative graphs of the most highly expressed miRs in the respective groups with miR-224-5p and miR-222-3p highly expressed only in the Low CFR group and not among the highest expressed miRNA in the High CFR group. Whereas the **d** is a comparative graph of the significant increase of the miR-224-5p in the Low CFR group through a Mann–Whitney plot

The q-PCR based micro-RNA profiling on EVs isolated from patient plasma samples to identify miRNAs contributing to microvascular dysfunction, revealed that, out of the 190 unique micro-RNAs in the assay, miR-224-5p and miR-222-3p (Fig. 1a) were significantly higher expressed in the low CFR group with p values of < 0.000001 and 0.000055 respectively. miR-133b (relative expression = 47.93), miR-31-5p (relative expression = 45.59), While miR-500a-5p (relative expression = 38.12) and miR-204-5p relative expression = 25.08), were included among the 190 miRNAs we assessed (sup.1), that were highly expressed in both the high and low CFR group (Fig. 1b, c), miR-224-5p and miR-222-3p remained the most statistically differentially expressed miRs in the Low CFR group (Fig. 1b). The miR-224-5p negatively correlated to Coronary Flow Reserve and was observed to be significantly present in the Low CFR group (Fig. 1d). To better understand this relationship CFR was subjected to a regression model dependent on the miR-224-5p. A significant regression equation was found (F = (1,68) = 5.820, *p* < 0.019) with an R2 of 0.079. The equation showed that CFR decreased -0.003-fold for every unit increase in the miR-224-5p. However, in our low CFR group, the median fold change for miR-224-5p was 14.83 for the miR. Hence, CFR decreased -0.045-fold for every relative fold change in the miR-224-5p. The independent relationship of the miR to CFR as well as its significantly differential expression in the LCFR group led us to further investigate the role of miR-224-5p.

### Origin of miR-224-5p containing EVs

**Fig. 2:**
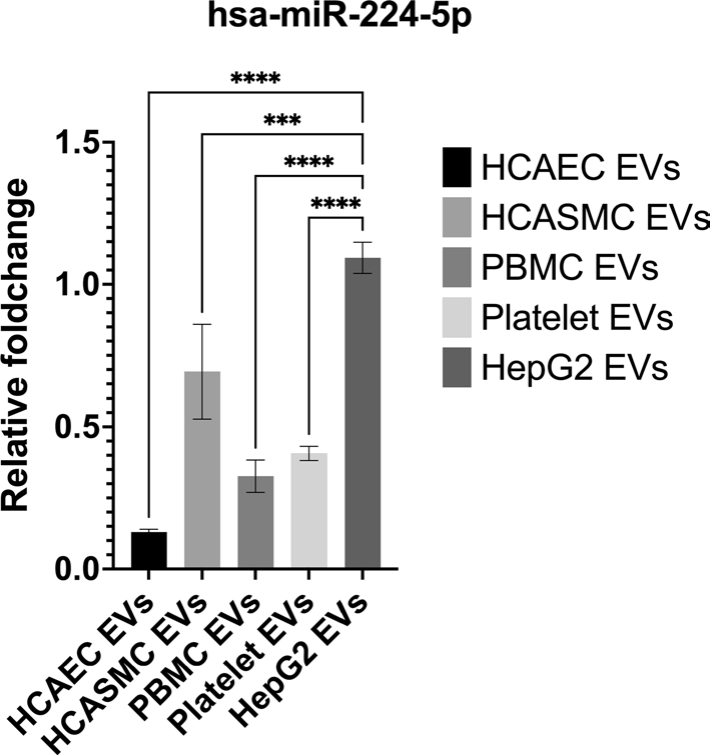
The bar graph presents the mean relative fold change of miR-224-5p in EVs from endothelial, smooth muscle, leukocytes, platelets as well as HepG2 cells compared to the reference gene; with HepG2 cell-released EV contain the highest expression of miR-224-5p

Literature survey indicated the origin of miR-224-5p from liver cells and not of platelet, leukocyte, smooth muscle or endothelial (EC) origin. The correlation between the miR-224-5p and CFR, indicated a possible intercellular communication via EVs containing the miR and ECs. The miR-224-5p had been previously studied in hepatocellular carcinomas and metabolic syndromes related to liver cells [33]. We, therefore, wanted to explore if the observed EVs were of hepatic or cardiovascular origin. We showed that upon inflammatory stimulus (TNF-alpha 10 ng/µl) HepG2 cells released more EVs containing miR-224-5p (Fig. 2), compared to endothelial, platelet, leukocyte, and smooth muscle cell types (n = 3).

### Liver generated EVs are present in the ProFlow cohort

**Fig. 3:**
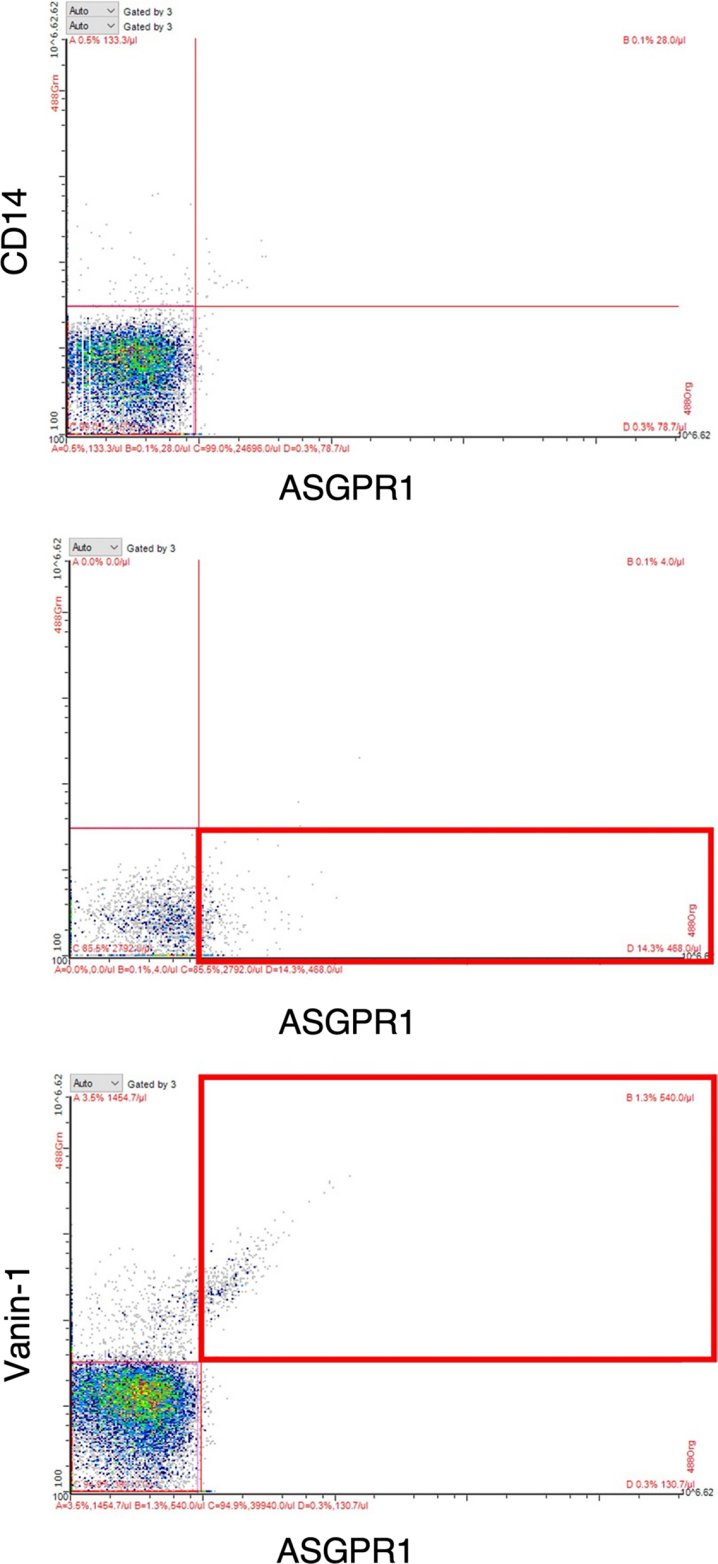
The FCM plot shows **a** the presence of Liver specific EVs stained by ASGPR-1 and CD14 surface markers. **b** Plots the substantial presence of Liver-specific EVs from HepG2 cells. **c** Whereas the last panel depicts the co-staining of Liver EVs with Vanin-1 required to enter the endothelial cell barrier

We further found the presence of liver EVs in plasma from the PROFLOW cohort patients to support our in-vitro findings. Asialoglycoprotein receptor-1 (ASGPR-1) is a liver-specific surface marker highly expressed on the hepatocellular membrane during inflammation [34]. As seen on Fig. 3, liver EVs, based on the ASGPR1 expression, are present in the circulation of the patients from the PROFLOW cohort. As ASGPR1 can also be present on monocytic lineage, a co-staining with CD14 monocyte marker was performed [35]. The analysis revealed that the majority of ASGPR1 + EVs are CD14- and thus derived from the liver. Additional staining confirmed that ASGPR1 is substantially expressed on the liver cancer cell line HepG2 cells (Fig. 3B). Interestingly, our data also revealed that a large proportion of circulating ASGPR1 + EVs also present vanin-1 on the surface (Fig. 3c), an enzyme previously shown essential for the uptake of liver EVs by recipient endothelial cells [36].

### In-vitro transfer of EVs carrying miR-224-5p

**Fig. 4:**
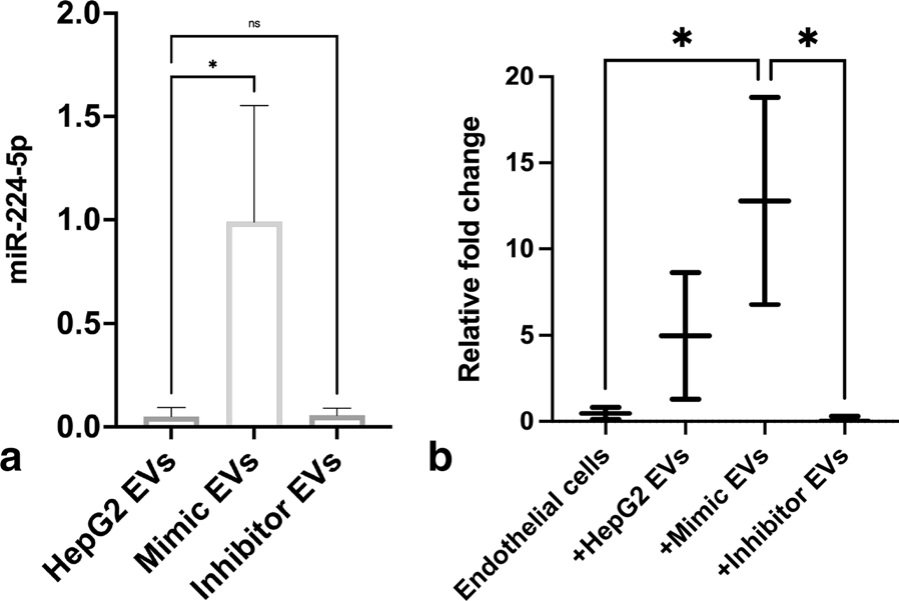
The bar graph (**a**) shows the transfection of the mimic for miR-224-5p in HepG2 EVs. Whereas the (**b**) represents the uptake of the EVs from HepG2 cells, Mimic, and Inhibitors for miR-224-5p cells by the HMVEC-1 cells with untreated cells as control

To study if EVs generated from liver cells with miR-224-5p can be transferred to ECs we transfected HepG2 cells with the miR-224-5p mimic and the inhibitor for the miR as a negative control. EVs were successfully generated from these cells upon treatment with TNF-alpha (10 ng/μl). The qPCR analysis showed a significant over-expression of miR-224-5p EVs generated from the mimic cells (Fig. 4a) compared to control cells.

Similarly, after 24 h of incubation with the mimic EVs, we observed an over-expression of miR-224-5p in the target cells i.e., HCMVEC cells. HCMVEC cells incubated with EVs from HepG2 cells, that naturally release miR-224-5p containing EVs, also showed an increase in the expression of miR-224-5p compared to the control ECs (Fig. 4b).

### Role of EVs with miR-224-5p in endothelial cells

After confirming that hepatic EVs, identified by the presence of miR-224-5p, are taken up by HCMVEC, we investigated its effect on viability and cell density. We observed that EVs with inhibitor for miR-224-5p resulted in significant decrease in metabolic activity and apoptosis in endothelial cells. There was no significant change in cell density, or metabolic activity, in ECs containing mimic and HepG2 EVs (Additional file 1: Fig. S2a, b).

### Activation of adhesion molecules by hepatic EVs

**Fig. 5:**
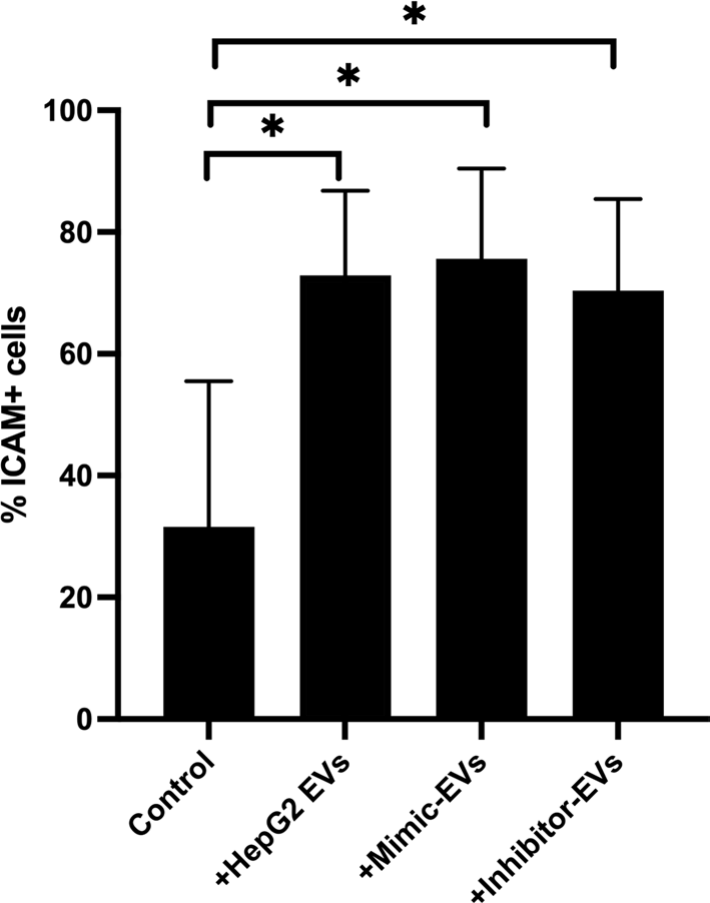
Shows a bar graph with elevated expression of surface ICAM-1 on HCMVEC cells when incubated with hepatic EVs

The elevated levels of miR-224-5p in our risk-group and the previous results suggested that the miR-224-5p probably functions as a marker for hepatic EVs in the circulation. We, hence, wanted to observe the effects of hepatic EVs identified by miR-224-5p on ICAM-1, known to mediate inflammatory responses at the endothelial level. We saw that the miR-224-5p content of the EVs per se, had no effect on endothelial ICAM-1. However, the presence of HepG2 EVs, alone increased the surface expression of ICAM-1 significantly by approximately 75% (Fig. 5).

## Discussion

In an effort to characterize CAD at the molecular level, we subjected EVs from a patient cohort, categorized by their CFR ratio, to a q-PCR based micro-RNA profiling. The patients of the used PROFLOW cohort were differentiated by their CFR ratio which indicates endothelial dysfunction and also predicts the development of MACE. Impaired cardiovascular function is a result of endothelial and microvascular dysfunction including inflammation, neovascularization, and fibrosis [37]. While it is known that presence of EVs increases with adverse conditions including the pathophysiology involved in the development of CAD [11] the importance of the EV miRNA content in this relationship is not well-characterized. We decided to do a micro-RNA profiling of EVs isolated from the PROFLOW cohort which resulted in identification of miR-224-5p as being inversely related to CFR and thus a putative indicator of disease. miR-224-5p has been primarily described as a hepatomiR, and its expression is elevated by the NFkB inflammatory pathway activated by TNF-alpha in Hepatocellular Carcinoma Cells [33]. The most abundant hepatomiR, miR-122-5p, known to play a crucial role in the development of atherosclerotic lesions [38] and known to be released in EVs [39, 40], was also included in the screening panel. There was, however, no significant difference in miR-122-5p between the two CFR groups. The supposed hepatic origin implored us to correlate the miRs to the liver function characteristics (ASAT and ALAT) of the cohort. Mir-122-5p showed a weak correlation to ALAT (sup.) but no correlation to ASAT and CFR. Whereas the miR-224-5p showed an independent and significant relationship to CFR. Since this study is focused on the development of MACE determined by CFR, we concentrated on miR-224-5p in the rest of this investigation.

We, therefore, focused on validating the origin of miR-224-5p. We demonstrated that stimulation of hepatic HepG2 cells with TNF-alpha led to an increase in a release of EVs containing miR-224-5p. The increase was higher for the liver derived HepG2 cells than for cells of endothelial, platelet, leukocyte, or smooth muscle ancestry. The origin was further validated in circulating plasma of patients by showing the presence of liver-specific (ASGPR-1+) EVs.

The next question was if hepatic EVs are able to cross the endothelial barrier. Vannin-1 has been reported to be required for permeabilization of the endothelial barrier during inflammation [36]. A flow cytometric approach, confirmed the co-staining of hepatic EVs (ASGPR-1+) with Vannin-1, thus showing that hepatic EVs can potentially permeate the endothelial barrier to deliver the miR-224-5p.

After demonstrating that EVs indeed can deliver miR-224-5p into HCMVECs we investigated its effect on pathways potentially involved in cardiovascular disease. Central to endothelial function/dysfunction are parameters reflecting viability and metabolism which is why we next investigated the effects of miR-224-5p on crystal violet staining and the CCK assay on HCMVEC. The results involving the miR-224-5p mimic and inhibitor showed that while there was no significant change in the endothelial cells to the presence of the miR, the absence of miR-224-5p led to a decrease in metabolic activity furthering apoptosis. This indicated that miR-224-5p probably is involved in maintaining homeostasis, as the lack of the miR-224-5p led to cell death. A possible mechanism for this is likely to involve regulation of the TGF-beta pathway by modulation of the SMAD unit [41, 42].

We had previously studied the expression of ICAM-1 with respect to platelet EVs using the same cohort [31], as ICAM-1 has been reported to play a crucial role in the pathophysiology of cardiovascular diseases by activating inflammatory responses at the endothelial level [43–45]. Hence, we then decided to study the influence of miR-224-5-p on endothelial surface expression of ICAM-1.Although we observed no difference between cells treated with inhibitor and mimic EVs for miR-224-5-p, there was, on the other hand, a significant increase in ICAM-1 after treating the cells with hepatic EVs regardless of whether they contained miR-224-5p or not. Thus, indicating that perhaps miR-224-5p should be regarded as a CFR dependent marker of a subpopulation of hepatic EVs rather than a causative factor of endothelial dysfunction.

Research has shown that liver as an organ plays a crucial role in immunobiology of inflammatory responses [46]. It has been shown that non-alcoholic fatty liver disease (NFALD) has an independent association with CVD [47, 48]. Liver hepatokines have also been studied in association with CVD. Their secretion by the liver can directly affect parameters affecting cardiovascular health such as glucose, lipid metabolism and inflammation modulation [49]. The elevated proteins we observed i.e., ICAM-1 and the enzyme Vannin-1 are known to respond to inflammation involving TNF-alpha furthering diapedesis or leakiness of the vasculature leading to impaired coronary flow [36, 50]. A study has also demonstrated that circulating hepatic EVs alter the serum metabolome particularly arginine metabolites that regulates vascular function [51]. It has been reported that Arg-1 promotes vascular inflammation and senescence resulting in ICAM-1elevation [52–54]. Studies have also shown that hepatic EVs deliver Arg-1 to endothelial cells and thus enhances endothelial dysfunction [55]. Several, other possible pathways exist, that could explain the increased expression of ICAM-1, including inflammatory response induced by EVs carrying CXCL19, alleviation in TLR9 by hepatic EV-bound mitochondrial DNA as well as pro-coagulant hepatic EVs leading to systemic inflammation [56–60]. Similar to our finding, a study showed subpopulation of EVs triggered by TNF-alpha increased the expression of ICAM-1 [61].

Factors such as age, dyslipidemia, smoking as well as hypertension have already been shown to impact development of MACE in the ProFlow cohort. Platelet EVs containing proteins such as BAFF have been shown in the same cohort to aid endothelial dysfunction in patients with Low CFR. We also observed elevated arterial stiffness in the patients with Low CFR in our cohort (sup.). We, hence, suggest that an overall inflammatory response led to a crosstalk between the liver and the cardiovascular system through EVs. The liver releases a subpopulation of vesicles identified by miR-224-5p in response to inflammation contributing to endothelial dysfunction. Other cytokines, proteins and miRs could have possible other functions reflecting the biological state of our cohort. The finding of miR-224-5p, in correlation to the Low-CFR group, and our investigation into its biological function, has led to the miR’s designation as a marker for a subpopulation of circulating hepatic EVs in patients with low CFR. It also demonstrates the contribution of hepatic EVs in the elevation of ICAM-1. Thus, highlighting a crosstalk between the liver and the cardiovascular systems contributing to endothelial dysfunction through circulating EVs.

## Supplementary Information

**Additional file 1**.** Sup.table.1**. Median relative fold change of all the 190 micro-RNAs. Sup.fig.1. Relationship of ALAT to miR-122-5p.** Sup.fig.2**. Bar graphs of the cell viability and cell density assays.** Sup.fig.3**. Bar graph of the Augmentation Index measured between the two CFR groups.** Sup.fig.4, 5 & 6**. Reproduced FCM plots for EVs with markers ASGPR-1,CD14 and Vannin-1. Detail description of antibodies and cell lines.

## Data Availability

All data generated or analysed during this study are included in this published article and its Additional files.

## Abbreviations

CFR: Coronary flow reserve
EVs: Extracellular vesicles
miRNA: Micro-RNA
PROFLOW: Prospective evaluation of coronary flow reserve
