# Supplementary material for: Increased expression of miR-224-5p in circulating extracellular vesicles of patients with reduced coronary flow reserve
Source: BMC Cardiovasc Disord. 2022 Jul 18;22:321. doi: 10.1186/s12872-022-02756-w (PMC9290204; doi:10.1186/s12872-022-02756-w)
Supplement: Supplementary file 1 — Additional file 1. Sup.table.1. Median relative fold change of all the 190 micro-RNAs. Sup.fig.1. Relationship of ALAT to miR-122-5p. Sup.fig.2. Bar graphs of the cell viability and cell density assays. Sup.fig.3. Bar graph of the Augmentation Index measured between the two CFR groups. Sup.fig.4, 5 & 6. Reproduced FCM plots for EVs with markers ASGPR-1,CD14 and Vannin-1. Detail description of antibodies and cell lines. [file 12872_2022_2756_MOESM1_ESM.docx]

# Supplementary Data

Sup.Table.S1: Median relative fold change of micro-RNAs between the two CFR group including the hepatic miR-122.

| miRNA | HCFR | LCFR |
| --- | --- | --- |
| hsa-let-7a-5p | 1,076256671 | 0,708938913 |
| hsa-let-7b-3p | 1,365946757 | 2,557129173 |
| hsa-let-7b-5p | 1,038287578 | 1,052992544 |
| hsa-let-7c-5p | 15,76878939 | 15,76878939 |
| hsa-let-7d-3p | 1,087490742 | 0,958638356 |
| hsa-let-7d-5p | 1,300635402 | 1,139200892 |
| hsa-let-7e-5p | 0,965154603 | 0,62967332 |
| hsa-let-7f-5p | 0,85027416 | 0,559523913 |
| hsa-let-7g-5p | 1,155487911 | 1,006397063 |
| hsa-let-7i-3p | 1,632962605 | 1,664382834 |
| hsa-miR-16-5p | 1,289255508 | 1,903399754 |
| hsa-let-7i-5p | 1,25962998 | 0,925036701 |
| hsa-miR-1-3p | 0,971268012 | 0,76763946 |
| hsa-miR-101-3p | 1,057501858 | 1,411281268 |
| hsa-miR-103a-3p | 1,174874795 | 1,740500425 |
| hsa-miR-106a-5p | 1,082975046 | 1,082975046 |
| hsa-miR-106b-3p | 1,517654992 | 2,179282734 |
| hsa-miR-106b-5p | 0,964630366 | 1,03674319 |
| hsa-miR-107 | 1,573883884 | 1,419747745 |
| hsa-miR-10a-5p | 1,761631865 | 1,29230961 |
| hsa-miR-10b-5p | 12,41489367 | 12,41489367 |
| hsa-miR-122-5p | 1,252707772 | 1,167896535 |
| hsa-miR-125a-5p | 1,100871167 | 1,106886287 |
| hsa-miR-125b-5p | 1,264989776 | 1,414924769 |
| hsa-miR-126-3p | 1,037165155 | 1,467134754 |
| hsa-miR-127-3p | 1,505715406 | 1,189921384 |
| hsa-miR-128-3p | 1,43399693 | 1,351210681 |
| hsa-miR-130a-3p | 1,096571589 | 1,096571589 |
| hsa-miR-130b-3p | 1,315082068 | 1,398175862 |
| hsa-miR-132-3p | 1,532973791 | 2,209077515 |
| hsa-miR-133a-3p | 1,597703833 | 3,166743463 |
| hsa-miR-133b | 68,16692502 | 68,16692502 |
| hsa-miR-136-5p | 4,25748073 | 4,25748073 |
| hsa-miR-139-5p | 1,078480432 | 0,850570171 |
| hsa-miR-140-3p | 1,193450413 | 1,205436128 |
| hsa-miR-140-5p | 1,172898343 | 1,306765254 |
| hsa-miR-141-3p | 8,016652841 | 8,016652841 |
| hsa-miR-142-3p | 1,33520398 | 1,664423059 |
| hsa-miR-142-5p | 0,935531141 | 0,800519618 |
| hsa-miR-143-3p | 1,442928687 | 1,364671916 |
| hsa-miR-144-3p | 1,043208892 | 1,081830448 |
| hsa-miR-144-5p | 2,242332156 | 2,242332156 |
| hsa-miR-145-5p | 1,395318383 | 1,283944206 |
| hsa-miR-146a-5p | 1,117210664 | 1,025783741 |
| hsa-miR-146b-5p | 1,439931319 | 1,439931319 |
| hsa-miR-148a-3p | 1,223583271 | 1,63059738 |
| hsa-miR-148b-3p | 1,146230353 | 1,191553608 |
| hsa-miR-150-5p | 0,986600695 | 0,677508942 |
| hsa-miR-151a-3p | 0,985290893 | 1,243367382 |
| hsa-miR-151a-5p | 1,178221896 | 0,961630228 |
| hsa-miR-152-3p | 1,022800691 | 0,96803097 |
| hsa-miR-154-5p | 6,058170358 | 3,289955174 |
| hsa-miR-155-5p | 1,067123099 | 1,666830642 |
| hsa-miR-15a-5p | 1,229514476 | 1,36435339 |
| hsa-miR-15b-3p | 1,330529041 | 1,330529041 |
| hsa-miR-15b-5p | 1,158292806 | 1,105350755 |
| hsa-miR-16-2-3p | 1,237322752 | 1,595730035 |
| hsa-miR-17-5p | 1,123146276 | 1,373637553 |
| hsa-miR-181a-5p | 1,112306549 | 0,864806405 |
| hsa-miR-182-5p | 2,008335086 | 1,825130977 |
| hsa-miR-185-5p | 1,098124733 | 1,372705536 |
| hsa-miR-186-5p | 1,079208195 | 1,10254983 |
| hsa-miR-18a-3p | 24,38308978 | 4,172872018 |
| hsa-miR-18a-5p | 1,135243193 | 1,24225528 |
| hsa-miR-18b-5p | 2,771546834 | 2,496566812 |
| hsa-miR-190a-5p | 2,009731986 | 1,969732886 |
| hsa-miR-191-5p | 1,137253718 | 1,097136757 |
| hsa-miR-192-5p | 1,435616951 | 1,747751516 |
| hsa-miR-193b-3p | 6,311574727 | 6,311574727 |
| hsa-miR-194-5p | 19,01412968 | 3,639077727 |
| hsa-miR-195-5p | 1,168787356 | 1,644607562 |
| hsa-miR-197-3p | 0,973333115 | 0,914199292 |
| hsa-miR-199a-3p_hsa-miR-199b-3p | 1,064035167 | 1,016454639 |
| hsa-miR-199a-5p | 0,915099168 | 0,917324607 |
| hsa-miR-19a-3p | 0,829741141 | 0,735500607 |
| hsa-miR-19b-3p | 1,004893029 | 1,219778009 |
| hsa-miR-200a-3p | 1,422077411 | 1,632254521 |
| hsa-miR-200c-3p | 4,64925643 | 4,64925643 |
| hsa-miR-204-5p | 39,89123822 | 39,89123822 |
| hsa-miR-205-5p | 1,000693387 | 1,000693387 |
| hsa-miR-208a-3p | 1,484523571 | 0,719929953 |
| hsa-miR-20a-3p | 2,211461307 | 2,211461307 |
| hsa-miR-20a-5p | 1,123524895 | 1,159353808 |
| hsa-miR-20b-5p | 1,016118312 | 1,500682327 |
| hsa-miR-21-3p | 1,18967942 | 1,386576365 |
| hsa-miR-21-5p | 1,182734216 | 1,71728474 |
| hsa-miR-210-3p | 11,36873635 | 3,162199631 |
| hsa-miR-2110 | 1,02533029 | 1,164544239 |
| hsa-miR-215-5p | 4,143931054 | 4,143931054 |
| hsa-miR-22-3p | 1,133931139 | 1,28905612 |
| hsa-miR-22-5p | 1,504233021 | 1,570291168 |
| hsa-miR-221-3p | 1,036503758 | 0,729816734 |
| hsa-miR-222-3p | 1,060213534 | 1,272657191 |
| hsa-miR-223-3p | 1,197751904 | 1,477857348 |
| hsa-miR-223-5p | 2,097837449 | 1,393842496 |
| hsa-miR-224-5p | 1,278194626 | 2,083487359 |
| hsa-miR-23a-3p | 1,035047907 | 1,002767618 |
| hsa-miR-23b-3p | 1,143628606 | 1,046004391 |
| hsa-miR-24-3p | 1,013259091 | 1,445308418 |
| hsa-miR-25-3p | 0,987278603 | 1,048628924 |
| hsa-miR-26a-5p | 1,190857135 | 1,441614887 |
| hsa-miR-26b-5p | 1,171250697 | 0,786217292 |
| hsa-miR-27a-3p | 0,940828142 | 0,9844999 |
| hsa-miR-27b-3p | 1,096945427 | 1,759925846 |
| hsa-miR-28-3p | 0,971644242 | 1,681120008 |
| hsa-miR-28-5p | 1,269356056 | 1,550795726 |
| hsa-miR-296-5p | 21,06827075 | 21,06827075 |
| hsa-miR-29a-3p | 0,998405332 | 0,950000383 |
| hsa-miR-29a-5p | 2,188587403 | 2,188587403 |
| hsa-miR-29b-2-5p | 4,870264075 | 13,82223933 |
| hsa-miR-29b-3p | 1,114220417 | 1,240170269 |
| hsa-miR-29c-3p | 1,139413535 | 1,161571513 |
| hsa-miR-301a-3p | 1,403141201 | 1,153617983 |
| hsa-miR-301b-3p | 6,639156685 | 6,639156685 |
| hsa-miR-30a-5p | 1,845484985 | 1,845484985 |
| hsa-miR-30b-5p | 1,026427537 | 1,067399438 |
| hsa-miR-30c-5p | 1,020320346 | 1,105081547 |
| hsa-miR-30d-5p | 0,850266684 | 1,083275804 |
| hsa-miR-30e-3p | 1,076242452 | 0,852634892 |
| hsa-miR-30e-5p | 1,093535457 | 1,093535457 |
| hsa-miR-31-5p | 69,11849983 | 69,11849983 |
| hsa-miR-32-5p | 1,174461971 | 1,174461971 |
| hsa-miR-320a | 1,113050843 | 0,82322281 |
| hsa-miR-320b | 1,247970008 | 1,244514045 |
| hsa-miR-320e | 1,02523071 | 1,143824758 |
| hsa-miR-324-3p | 0,736623843 | 0,735093668 |
| hsa-miR-324-5p | 1,200884003 | 1,254049977 |
| hsa-miR-326 | 1,382711418 | 1,84483939 |
| hsa-miR-328-3p | 1,130922756 | 1,328685814 |
| hsa-miR-331-3p | 0,996936048 | 0,839149637 |
| hsa-miR-335-5p | 0,687770909 | 0,677375669 |
| hsa-miR-338-3p | 1,934534392 | 3,060565742 |
| hsa-miR-339-3p | 1,331463128 | 1,185384306 |
| hsa-miR-339-5p | 1,095824665 | 1,46112335 |
| hsa-miR-33a-5p | 1,871246996 | 1,994462503 |
| hsa-miR-342-3p | 1,095090352 | 1,143577743 |
| hsa-miR-346 | 9,835510938 | 9,835510938 |
| hsa-miR-34a-5p | 2,733931423 | 2,960099442 |
| hsa-miR-361-3p | 1,662697691 | 2,321764866 |
| hsa-miR-363-3p | 1,188383105 | 1,703363369 |
| hsa-miR-365a-3p_hsa-miR-365b-3p | 1,80000386 | 1,80000386 |
| hsa-miR-373-3p | 5,971113533 | 5,971113533 |
| hsa-miR-373-5p |  |  |
| hsa-miR-374a-5p | 0,856676475 | 0,836677398 |
| hsa-miR-374b-5p | 0,913902578 | 1,445932642 |
| hsa-miR-375 | 0,649319301 | 0,649319301 |
| hsa-miR-376a-3p | 1,262748449 | 2,11696879 |
| hsa-miR-378a-3p | 2,848100391 | 2,187070915 |
| hsa-miR-382-5p | 1,582105825 | 2,33269874 |
| hsa-miR-409-3p | 1,285206337 | 1,603906572 |
| hsa-miR-421 | 1,183451022 | 0,862143545 |
| hsa-miR-423-3p | 1,037846282 | 1,01517282 |
| hsa-miR-16-5p | 1,082977387 | 1,562271891 |
| hsa-miR-423-5p | 1,223495389 | 0,76297183 |
| hsa-miR-424-5p | 1,322713658 | 1,699471987 |
| hsa-miR-425-3p | 1,176510864 | 1,006729047 |
| hsa-miR-425-5p | 1,0189395 | 0,976708529 |
| hsa-miR-451a | 1,162474186 | 0,987811115 |
| hsa-miR-483-3p | 2,126889819 | 2,322248631 |
| hsa-miR-483-5p | 11,80233166 | 2,170458744 |
| hsa-miR-484 | 1,065731388 | 0,977553323 |
| hsa-miR-485-3p | 1,387761513 | 2,066260543 |
| hsa-miR-486-5p | 1,020661335 | 0,871407297 |
| hsa-miR-495-3p | 1,379700247 | 2,035808646 |
| hsa-miR-497-5p | 1,260946458 | 1,429516513 |
| hsa-miR-500a-5p | 54,11662639 | 54,11662639 |
| hsa-miR-501-3p | 1,525201653 | 1,525201653 |
| hsa-miR-502-3p | 1,111108729 | 1,411275843 |
| hsa-miR-505-3p | 1,490710745 | 2,122846418 |
| hsa-miR-532-3p | 3,458148925 | 4,248636746 |
| hsa-miR-532-5p | 1,518582172 | 4,981591037 |
| hsa-miR-543 | 1,473247686 | 1,384853366 |
| hsa-miR-551a | 0,890692901 | 0,890692901 |
| hsa-miR-551b-3p | 1,25962998 | 1,25962998 |
| hsa-miR-574-3p | 1,032398535 | 1,032398535 |
| hsa-miR-584-5p | 1,088638558 | 0,683991312 |
| hsa-miR-590-5p | 2,491474831 | 2,491474831 |
| hsa-miR-605-5p | 31,8451111 | 31,8451111 |
| hsa-miR-629-5p | 17,16026587 | 17,16026587 |
| hsa-miR-652-3p | 1,501037531 | 1,165642277 |
| hsa-miR-660-5p | 1,357129666 | 1,028014016 |
| hsa-miR-766-3p | 1,028826708 | 0,749121195 |
| hsa-miR-885-5p | 7,225012081 | 7,225012081 |
| hsa-miR-92a-3p | 1,064108782 | 0,745032421 |
| hsa-miR-92b-3p | 1,326393782 | 1,234567352 |
| hsa-miR-93-3p | 5,715976559 | 5,715976559 |
| hsa-miR-93-5p | 1,063632673 | 1,437064792 |
| hsa-miR-95-3p | 16,46122733 | 16,46122733 |
| hsa-miR-99a-5p | 0,686342216 | 0,686342216 |
| hsa-miR-99b-5p | 1,137492933 | 1,248671606 |


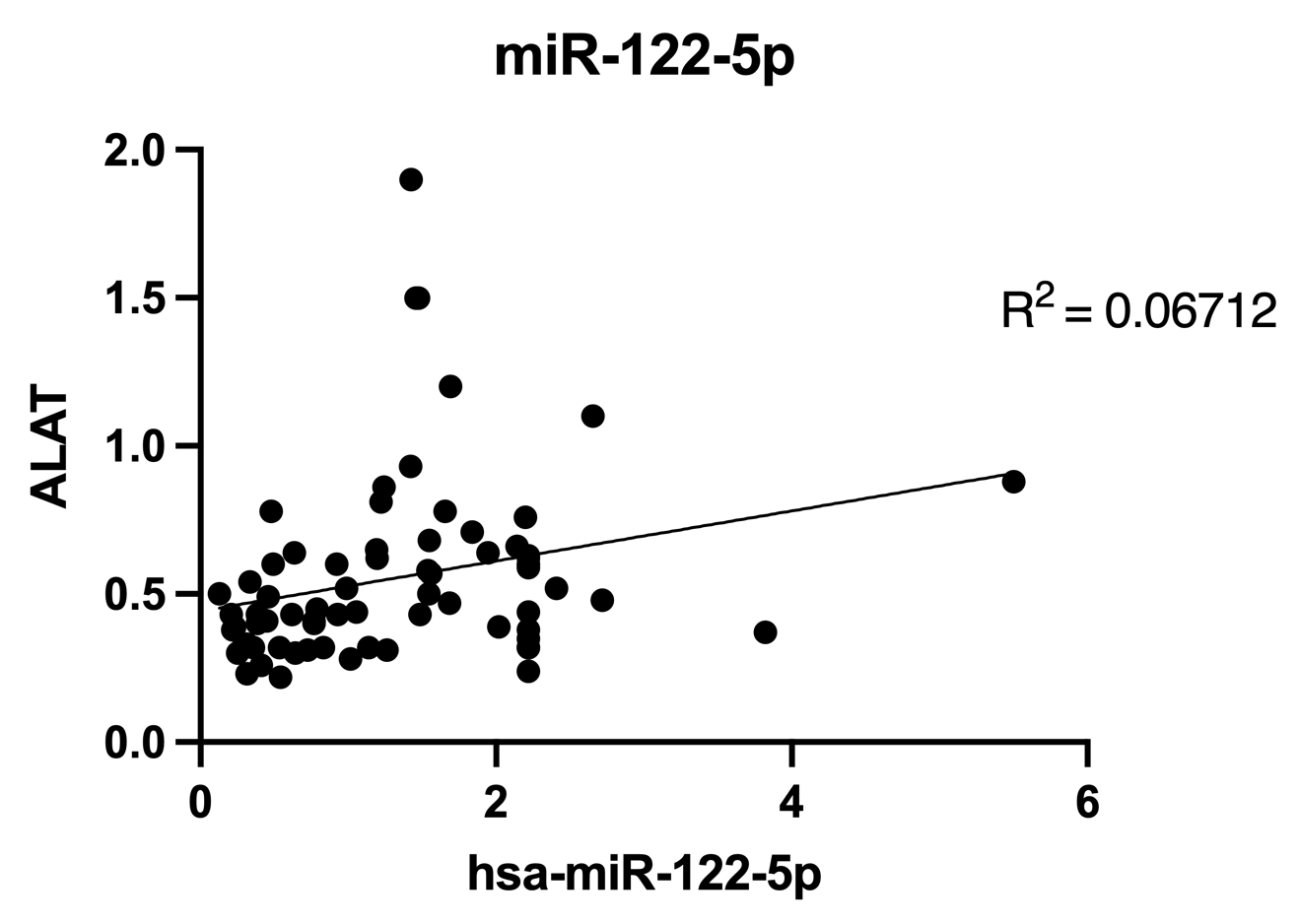


Sup.Fig.S1: The regression plot demonstrates the weak relationship of miR-122-5p to ALAT with r^2^=0.06.





Sup.Fig.S2: The Fig.S2.a & b show the significant decrease in viability and cell density when miR-224-5p is inhibited in HCMVEC cells by EVs while demonstrating no effect of the miR when present by CCK-8 and Crystal-violet assay.


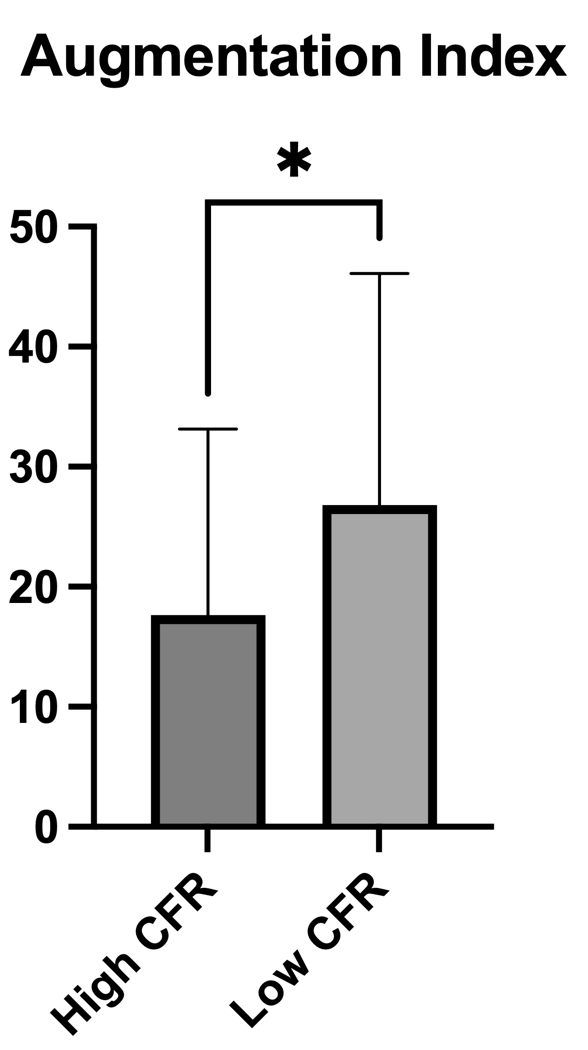


Sup.fig.S3: The bar plot shows the significant increase in arterial stiffness in patients from the Low CFR group demonstrated by Augmentation Index.

**
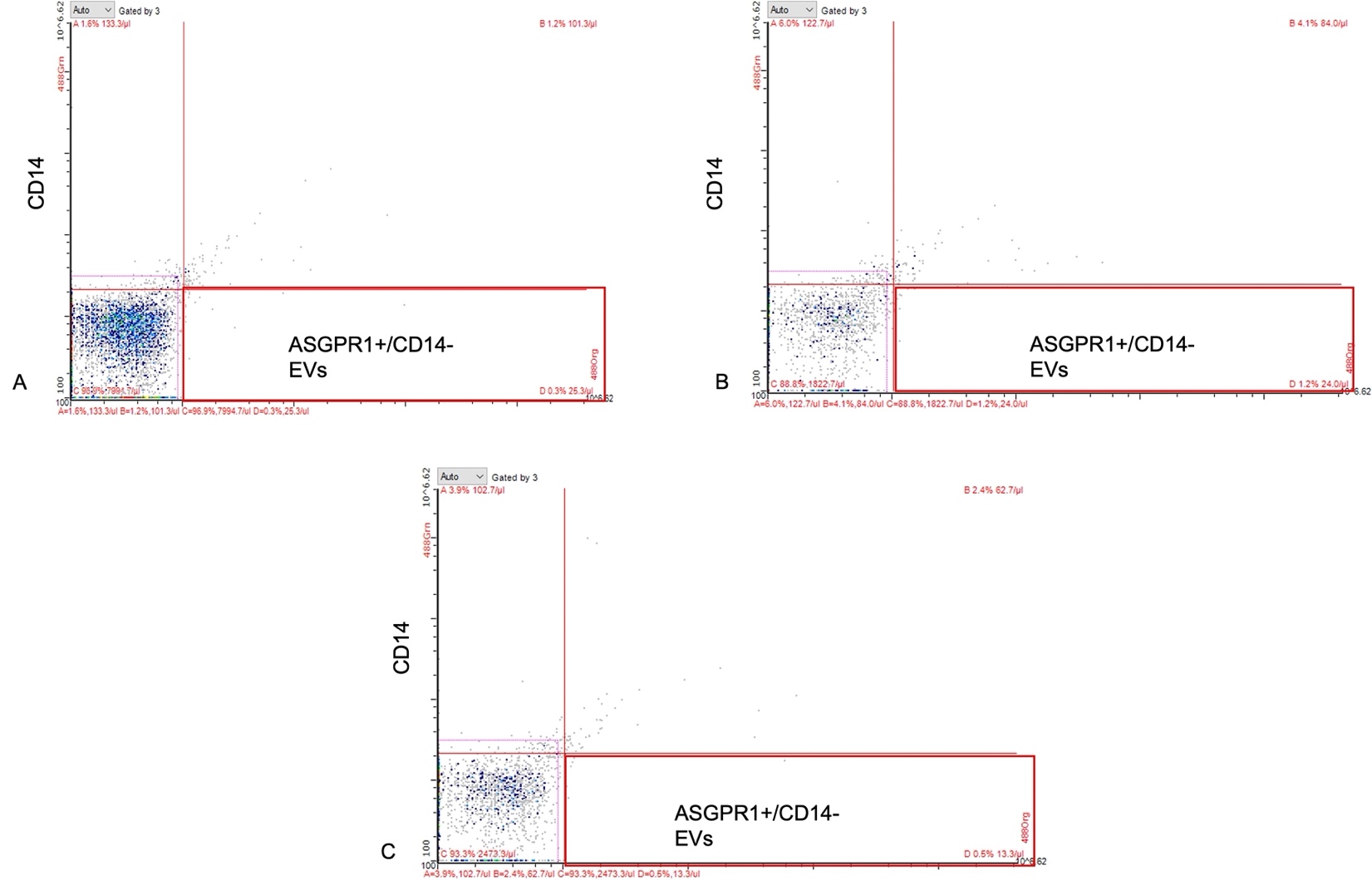
**

Sup.fig.S4: The flow Cytometry datagrams demonstrate the presence of ASGPR1+/CD14- EVs in 3 different plasma samples

**
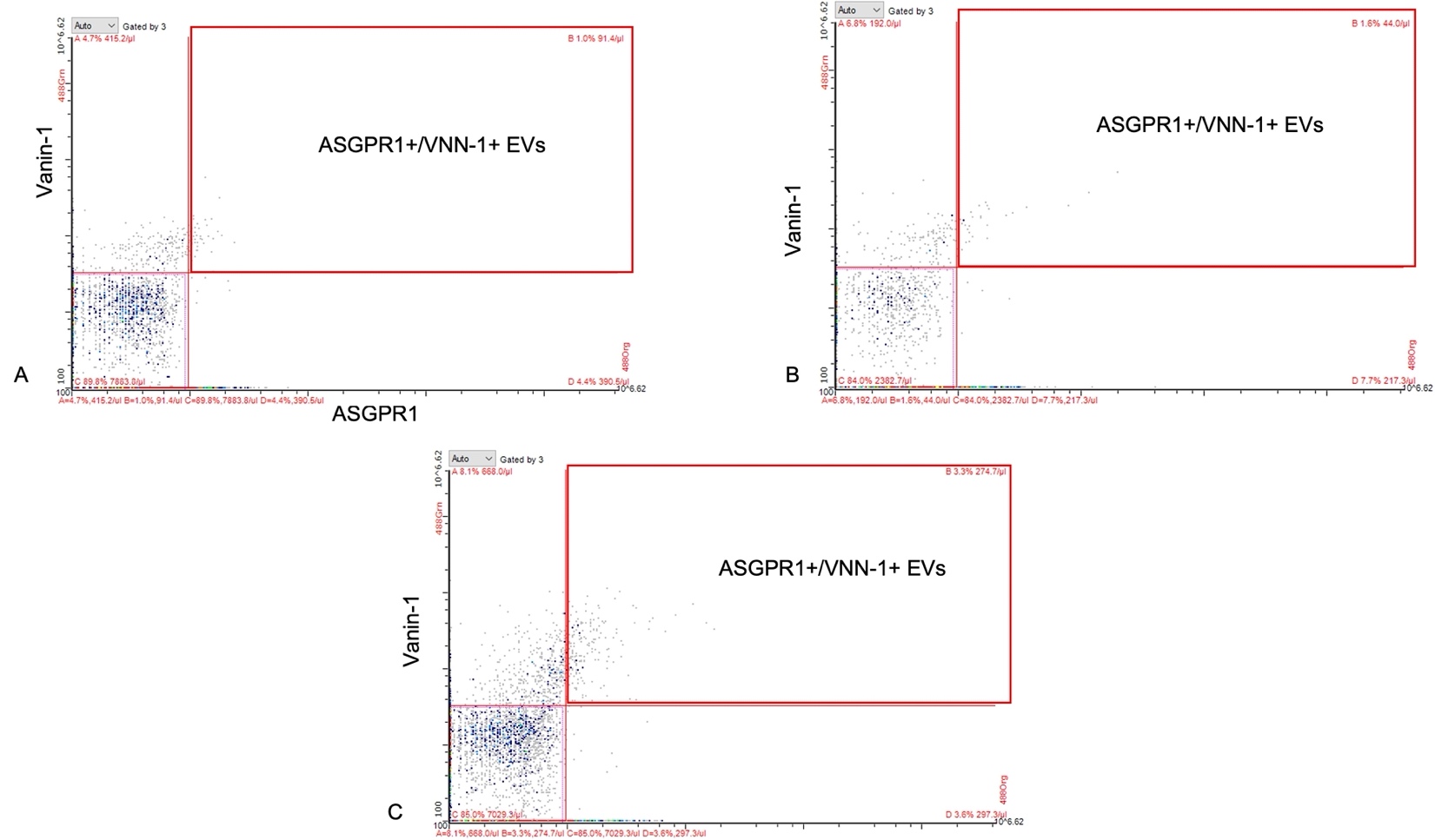
**

Sup.fig.S5: The flow Cytometry datagrams demonstrate the presence of ASGPR1+/Vanin-1+ EVs in 3 different plasma samples

**
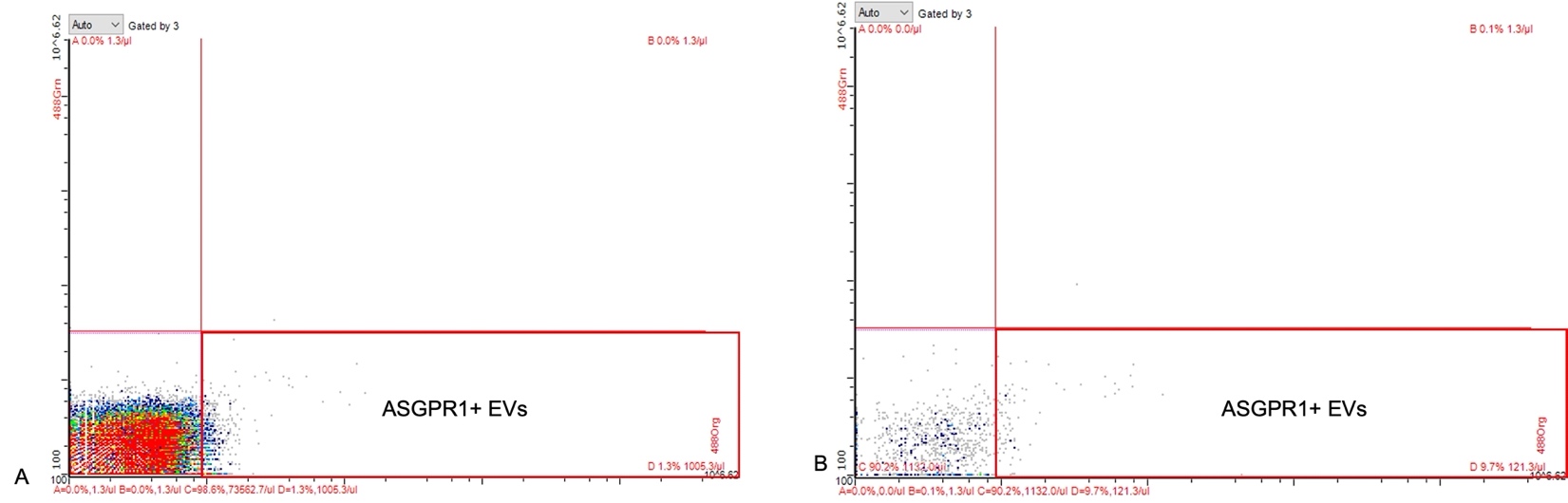
**

Sup.fig.S6: The flow Cytometry datagrams demonstrate the presence of ASGPR1(1)+ EVs in 2 different plasma samples

**Antibodies**

| **Target antigen** | **Vendor or Source** | **Catalog #** | **Working concentration** | **Lot # (preferred but not required)** | **Persistent ID / URL** |
| --- | --- | --- | --- | --- | --- |
| ASGPR-1 | BD Biosciences | 563655 | 1:100 |  | <https://www.bdbiosciences.com/en-us/products/reagents/flow-cytometry-reagents/research-reagents/single-color-antibodies-ruo/pe-mouse-anti-asgpr-1.563655> |
| Vanin-1 | Biorbyt | orb222619 | 1:100 |  | <https://www.biorbyt.com/vnn1-antibody-fitc-orb222619.html> |
| CD-14 | BD Biosciences | 555397 | 1:100 |  | <https://www.bdbiosciences.com/en-us/products/reagents/flow-cytometry-reagents/research-reagents/single-color-antibodies-ruo/fitc-mouse-anti-human-cd14.555397> |

**Cultured Cells**

| **Name** | **Vendor or Source** | **Sex (F, M, or unknown)** | **Persistent ID / URL** |
| --- | --- | --- | --- |
| HMEC-1 | ATCC | unknown | <https://www.atcc.org/products/crl-3243> |
| HCAEC | PromoCell | unknown | <https://promocell.com/product/human-coronary-artery-endothelial-cells-hcaec/> |
| HCMVEC | Lonza | unknown | [https://bioscience.lonza.com/lonza_bs/US/en/Primary-and-Stem-Cells/p/000000000000185434/HMVEC-C-–-Human-Cardiac-Microvascular-Endothelial-Cells](https://bioscience.lonza.com/lonza_bs/US/en/Primary-and-Stem-Cells/p/000000000000185434/HMVEC-C-%E2%80%93-Human-Cardiac-Microvascular-Endothelial-Cells) |
| HepG2 | Sigma-Aldrich | unknown | <https://www.atcc.org/products/hb-8065> |
| HCASMC | Gibco | unknown | <https://www.fishersci.ca/shop/products/human-coronary-artery-smooth-muscle-cells-hcasmc/c0175c> |

**MiRCURY PCR Assay**

| **Name** | **Source / Repository** | **Persistent ID / URL** |
| --- | --- | --- |
| U6 snRNA miRCURY LNA miRNA PCR Assay  GeneGlobe Id: YP00203907  Catalog Number: 339306 | Qiagen | <https://geneglobe.qiagen.com/us/product-groups/mircury-lna-mirna-pcr-assays?q=U6> |
| hsa-miR-224-5p miRCURY LNA miRNA PCR Assay  GeneGlobe Id: YP02119313  Catalog Number: 339306 | Qiagen | <https://geneglobe.qiagen.com/us/product-groups/mircury-lna-mirna-pcr-assays?q=hsa-miR-224-5p> |
| hsa-miR-222-3p miRCURY LNA miRNA PCR Assay  GeneGlobe Id: YP00204551  Catalog Number: 339306 | Qiagen | <https://geneglobe.qiagen.com/us/product-groups/mircury-lna-mirna-pcr-assays?q=hsa-miR-222-3p> |
| Product Name: cel-miR-39-3p miRCURY LNA miRNA PCR Assay | Qiagen | <https://geneglobe.qiagen.com/us/product-groups/mircury-lna-mirna-pcr-assays?q=cel-miR-39> |
